# Biological Evaluation of Acellular Cartilaginous and Dermal Matrixes as Tissue Engineering Scaffolds for Cartilage Regeneration

**DOI:** 10.3389/fcell.2020.624337

**Published:** 2021-01-11

**Authors:** Yahui Wang, Yong Xu, Guangdong Zhou, Yu Liu, Yilin Cao

**Affiliations:** ^1^Research Institute of Plastic Surgery, Wei Fang Medical College, Weifang, China; ^2^National Tissue Engineering Center of China, Shanghai, China; ^3^Shanghai Key Laboratory of Tissue Engineering, Department of Plastic and Reconstructive Surgery, Shanghai 9th People’s Hospital, Shanghai Stem Cell Institute, Shanghai Jiao Tong University School of Medicine, Shanghai, China; ^4^Department of Thoracic Surgery, Shanghai Pulmonary Hospital, Tongji University School of Medicine, Shanghai, China

**Keywords:** acellular cartilaginous matrix, acellular dermal matrix, tissue engineering, cartilage regeneration, immune responses

## Abstract

An acellular matrix (AM) as a kind of natural biomaterial is gaining increasing attention in tissue engineering applications. An acellular cartilaginous matrix (ACM) and acellular dermal matrix (ADM) are two kinds of the most widely used AMs in cartilage tissue engineering. However, there is still debate over which of these AMs achieves optimal cartilage regeneration, especially in immunocompetent large animals. In the current study, we fabricated porous ADM and ACM scaffolds by a freeze-drying method and confirmed that ADM had a larger pore size than ACM. By recolonization with goat auricular chondrocytes and *in vitro* culture, ADM scaffolds exhibited a higher cell adhesion rate, more homogeneous chondrocyte distribution, and neocartilage formation compared with ACM. Additionally, quantitative polymerase chain reaction (qPCR) indicated that expression of cartilage-related genes, including ACAN, COLIIA1, and SOX9, was significantly higher in the ADM group than the ACM group. Furthermore, after subcutaneous implantation in a goat, histological evaluation showed that ADM achieved more stable and matured cartilage compared with ACM, which was confirmed by quantitative data including the wet weight, volume, and contents of DNA, GAG, total collagen, and collagen II. Additionally, immunological assessment suggested that ADM evoked a low immune response compared with ACM as evidenced by qPCR and immunohistochemical analyses of CD3 and CD68, and TUNEL. Collectively, our results indicate that ADM is a more suitable AM for cartilage regeneration, which can be used for cartilage regeneration in immunocompetent large animals.

## Introduction

Cartilaginous defect repair is difficult because of the avascular nature and limited regeneration ability of cartilage *in situ* (Gomoll et al., 2010; Montgomery et al., 2014; Orth et al., 2020). Autogenous cartilage transplantation, allogenic cartilage transplantation, and artificial substitutes are therapeutic options. However, they have many shortcomings such as source limitations, immune rejection, transmission of exogenous diseases, foreign body reactions, and infection. In recent years, the development of cartilage tissue engineering has provided a new modality (Liu et al., 2020). Nevertheless, there is still a great challenge to generate mature and stable neocartilages in subcutaneous sites, such as the ear, nose, and eyelid, in immunocompetent individuals.

Biomaterial scaffolds play an important role in cartilage tissue engineering (Przekora, 2019; Lapomarda et al., 2020). They not only provide support to maintain the original shape, but also guide the regeneration of damaged cartilaginous tissue. Synthetic polymers (e.g., PLGA, PLA, and PGA) are often used as biomaterial scaffolds to generate cartilaginous tissue. However, because of the serious inflammatory response in immunocompetent animals, they are not ideal for cartilage regeneration and translation to clinical application (Rotter et al., 2005; Ceonzo et al., 2006; Asawa et al., 2012). Therefore, natural scaffolds such as an acellular matrix (AM) are gaining increasing attention for cartilage regeneration, because they provide an ideal extracellular matrix (ECM) and signals that facilitate cell attachment, migration, proliferation, and differentiation (Yang et al., 2008; Lammi et al., 2018).

AM is a kind of natural biomaterial with advantages including good histocompatibility, a satisfactory cellular adhesion rate, and suitable degradability to match the neo-tissue growth. An acellular cartilaginous matrix (ACM) and acellular dermal matrix (ADM) are two of the most widely used AMs in cartilage tissue engineering. ACM provides a proper microenvironment for chondrocyte growth and cartilage formation in theory because they retain most of the native cartilage-specific ECM structures and functional proteins (Choi et al., 2010; Schwarz et al., 2012, 2015; Kiyotake et al., 2016; Goldberg-Bockhorn et al., 2018; Zhang et al., 2018). Recently, accumulating studies have demonstrated that ADM is also able to generate cartilage tissue and has advantages over ACM, such as more sources and lower costs (Sherris and Oriel, 2011; Ma et al., 2013; Qi et al., 2014; Ye et al., 2016, 2018). However, few studies have been focused on which kind of AM material is more suitable for cartilage tissue engineering and no breakthroughs have been achieved in cartilage tissue engineering using AMs from a xenogeneic source in immunocompetent large animals. Consequently, it is of great importance to find a more appropriate AM as a scaffold material by comparing ACM and ADM that are suitable for cartilage regeneration in immunocompetent large animals.

In the current study, ACM and ADM were seeded with chondrocytes, cultured *in vitro*, and then subcutaneously implanted *in vivo* to investigate differences in cartilage regeneration by ACM and ADM as well as the outcomes of ACM and ADM from a xenogeneic source in subcutaneous sites of immunocompetent large animals. Such elucidation would provide insights to clarify the abilities of the two different AM materials to generate cartilage and thus provide a practical method for clinical translation of xenogeneic AM materials.

## Materials and Methods

### Preparation of ACM and ADM Scaffolds

ACM and ADM provided by JiangSu Unitrump Biomedical Technology Co., Ltd. (Nantong, Jiangsu) were derived from hyaline cartilage of hip joint and pig dermis, respectively. Both of cartilage and dermis tissues were carefully stripped of overlying soft tissue and the potential viruses were inactivated by ultraviolet irradiation. Then, a decellularization solution comprised of 2 mg/mL trypsin was combined with 4 mg/mL sodium dodecyl sulfate was used to remove the cells and cellular antigens in cartilage and dermis tissues. Thereafter, both the cartilage and dermis tissues were homogenized by high speed homogenizer. The ADM homogenate was crosslinked using 0.2% glutaraldehyde at PH 4.0–5.5, and followed with repeated lyophilization and rinse to achieve an ADM porous scaffold. In addition, the ACM suspension was mixed with ADM homogenate at a 7:3 ratio by weight and was crosslinked using 0.2% glutaraldehyde at PH 4.0–5.5, and followed with repeated lyophilization and rinse to achieve an ACM porous scaffold.

The microstructures of ACM and ADM scaffolds were examined by scanning electron microscopy (SEM; Philips XL-30, Amsterdam, Netherlands). The pore size was evaluated in SEM images by ImageJ software.

### *In vitro* Experiments

#### Isolation and Culture of Chondrocytes

This study was approved by the Weifang Medical University Ethics Committee. Three 8-month-old goats provided by Shanghai Jiagan Breeding Factory were used in this study. After intravenous anesthesia with 5% sodium pentobarbital (0.5 mL/kg), cartilage of 3 × 3 cm in size was harvested from an ear and transferred to a 50 mL centrifuge tube filled with 0.25% chloramphenicol. After removing the superfluous fibrous tissue and perichondrium, the cartilage tissue was dissected into 1 mm^3^ piece, washed in phosphate-buffered saline (PBS), and digested with 0.3% collagenase NB4 (Worthington biochemical Crop., Freehold, New Jersey, United States) for 8 h at 37°C. Then, the isolated cells were collected and cultured in Dulbecco’s modified Eagle’s medium (Gibco BRL, Grand Island, New York, United States) containing 10% fetal bovine serum (Gibco BRL) and 1% Antibiotic-Antimycotic (Gibco BRL) ^[32]^. Cells were passaged at >80% confluence. Passage 3 cells were used for experiments.

#### Preparation of Chondrocyte-Scaffold Constructs

A 5 mL suspension of chondrocytes at 1 × 10^8^ cells/mL was seeded in each scaffold, followed by 4 h of incubation at 37°C with 5% CO_2_ in 6-well plates. The constructs were then gently transferred to new 6-well plates. After 24 h of culture, the cell-scaffold constructs were gently rinsed with PBS to remove dead cells. The rinsing solution and cells remaining in the culture dish were collected and counted as N. The cell adhesion rate on the scaffolds was calculated by the following formula: (total cell number–*N*)/total cell number × 100%.

Cell-scaffold constructs were rinsed with PBS and fixed overnight at 4°C in 0.05% glutaraldehyde. After dehydration through a graded series of ethanol solutions, samples were critical point dried and examined by SEM to directly observe the attachment and distribution of chondrocytes and assess ECM synthesis on the scaffolds.

To determine cell viabilities in ACM and ADM scaffolds, chondrocytes were seeded at 25 × 10^6^/mL in ACM and ADM scaffolds. After 1, 4, and 7 days of culture, viability of the seeded cells was evaluated using the Live and Dead Cell Viability Assay (Invitrogen, United States), following the manufacturer’s instructions, under a confocal microscope (Nikon, Japan).

After 8 weeks of culture, samples were harvested for gross, histological, quantitative polymerase chain reaction (qPCR), and quantitative analyses.

### *In vivo* Experiments

Chondrocyte-scaffold constructs in ACM and ADM groups were implanted subcutaneously around the abdominal costal region of the goats. Samples were harvested at 1, 4, and 12 weeks post-implantation for gross, histological, immunohistochemical, quantitative, and qPCR evaluations.

### Histological and Immunohistochemical Analyses

Samples were fixed in 4% paraformaldehyde and embedded in paraffin for sectioning at 5 μm thicknesses and then mounted on glass slides. Sections were stained with hematoxylin and eosin (HE) and Safranin-O (SO).

For immunohistochemical analysis, terminal deoxynucleotidyl transferase biotin-dUTP nick end labeling (TUNEL) to detect apoptotic cells was performed using a TUNEL kit (Roche, Indianapolis, IN, United States) in accordance with the manufacturer’s instructions. A rabbit anti-human monoclonal antibody against collagen II (COL II) was used with a horseradish peroxidase (HRP)-conjugated anti-rabbit antibody (1:400 in PBS, Santa Cruz) as the secondary antibody. CD3 was detected using a rabbit anti-human CD3 monoclonal antibody (1:100 in PBS, Santa Cruz Biotechnology, Santa Cruz, CA, United States). CD68 was detected using a rabbit anti-human CD68 monoclonal antibody (1:1,000 in PBS, Santa Cruz Biotechnology). Color development was conducted with diaminobenzidine tetrahydrochloride (Santa Cruz Biotechnology).

### Quantitative Determination

Neocartilage samples from the various groups were weighed with an electronic balance. In addition, the neocartilage sample was immersed in 5 mL absolute ethanol and the change in volume was determined as the volume of the neocartilage sample. The contents of cartilage-specific matrices in engineered tissues were analyzed quantitatively.

Total glycosaminoglycan (GAG) content was analyzed by spectrophotometric microdetermination with dimethylmethylene blue. Briefly, total GAG was precipitated by guanidinium chloride solution (0.98 mol/L). After dissolving the GAG precipitate, the OD values were determined at 595 nm. A standard curve was established using chondroitin-4-sulfate, and total GAG was determined from the OD value correlating to the corresponding GAG amount in the standard curve.

The total collagen content was quantified by a hydroxyproline assay. Samples were prepared by alkaline hydrolysis and free hydroxyproline hydrolysates were assayed. Samples were prepared by alkaline hydrolysis, and free hydroxyproline hydrolyzates were assayed according to previously described methods (Reddy and Enwemeka, 1996). The hydroxyproline content was finally converted to total collagen content according to the mass ratio of 7.25 for collagen to hydroxyproline. The amount of collagen II was measured by an ELISA. DNA content was determined using a total DNA quantification assay (PicoGreen dsDNA assay, Invitrogen, United States).

### Quantitative Polymerase Chain Reaction (qPCR)

Expression of cartilage-related genes (ACAN, COL2A1, and SOX9) and inflammation-related genes (CD3 and CD68) was analyzed by qPCR. Total RNA was extracted with TRIzol reagent (Invitrogen), and reverse transcribed using Moloney murine leukemia virus Reverse Transcriptase (Invitrogen). qPCR was performed using a Fast Synergy Brands Green Master Kit and Light Cycler 480 system (Roche) in accordance with the manufacturer’s instructions. The results were analyzed using the comparative threshold cycle method and normalized to endogenous reference gene GAPDH. Results are reported as relative values to the mean gene expression of control ACM constructs cultured for 1 week *in vitro* or ACM constructs cultured for 1 week *in vivo*. All primer sequences are given in Table 1.

**TABLE 1:** Primer sequences of related genes.

Gene	Accession number	Primer sequence (5′–3′)
ACAN	XM_018066613.1	CAGAGGCAACCACAACAGACA AGCTGGGAAGGCATAAGCATG
COLIIA1	XM_018047868.1	GCATTGCCTACCTGGACGAAG TCACAGTCTCGCCCCACTTAC
SOX9	XM_018063905.1	AAGAACAAGCCGCACGTCAA CCGTTCTTCACCGACTTCCTC
CD3	XM_005689508.3	TTATCAGTGCCTCGCAACCG CTTTCGGCTCTTGCTCCAGTA
CD68	XM_005693517.3	AGCCCAGATTCAGATGCGAGT GATCCTGTTTGAATCCGAAGCT

### Statistical Analysis

Differences in quantitative data were analyzed using SPSS 23. A *p*-value of less than 0.05 was considered statistically significant.

## Results

### Biocompatibilities of ACM and ADM Scaffolds

**FIGURE 1:**
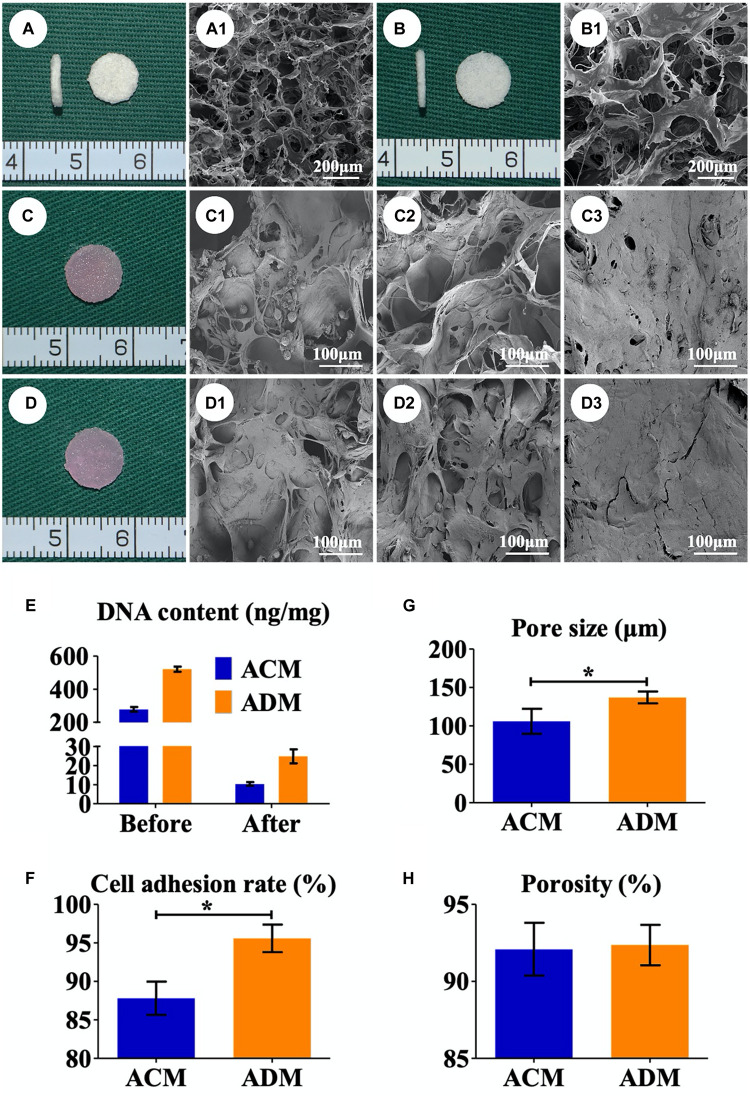
Preparation of *in vitro* ECs. Gross **(A)** and SEM **(A1)** images of the ACM scaffold. Gross **(B)** and SEM **(B1)** images of the ADM scaffold. Gross views immediately after chondrocytes were seeded onto ACM **(C)** and ADM **(D)**. SEM images of chondrocyte-ACM constructs after *in vitro* culture for 1 **(C1)**, 4 **(C2)**, and 7 **(C3)** days. SEM images of chondrocyte-ADM constructs after *in vitro* culture for 1 **(D1)**, 4 **(D2)**, and 7 **(D3)** days. The residual DNA content before and after decellularization in ACM and ADM scaffolds **(E)**. Pore size **(G)**, porosity **(H)**, and cell adhesion rate **(F)** of ACM and ADM scaffolds. EC, engineered cartilage. *P < 0.05.

**FIGURE 2:**
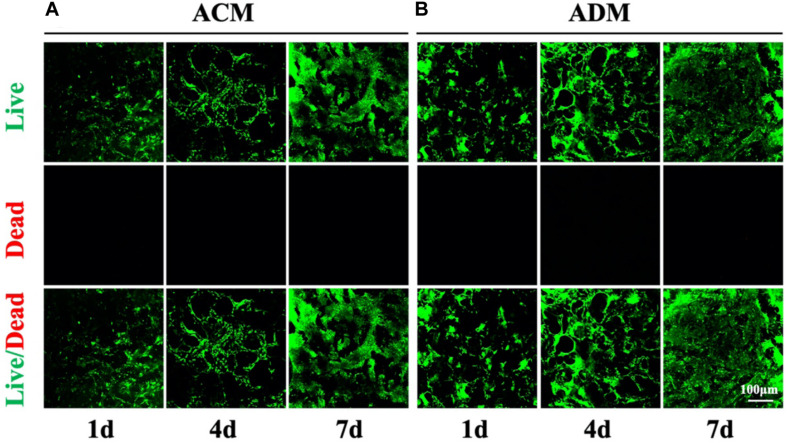
Cell viability of chondrocytes in ACM and ADM scaffolds. Live/dead staining of chondrocytes in ACM for 1, 4, and 7 days **(A)**. Live/dead staining of chondrocytes in ADM for 1, 4, and 7 days **(B)**.

A residual DNA content lower than 50 ng/mg was deemed as successful decellularization (Zhang et al., 2019). In the current study, we successfully prepared both ACM and ADM scaffolds with extremely low residual DNA content (10.34 ± 0.88 ng/mg for ACM and 24.80 ± 3.31 ng/mg for ADM) after decellularization process (Figure 1E). To evaluate the biocompatibilities of ACM and ADM scaffolds, auricular chondrocytes were seeded on ACM and ADM scaffolds. Both ACM (Figures 1A,A1) and ADM (Figures 1B,B1) scaffolds had three-dimensional porous structures with favorable interconnectivity and tremendous porosity (92.08 ± 1.54% for ACM and 92.36 ± 1.17% for ADM) (Figure 1H), whereas ADM had a higher average pore size than ACM (105.6 ± 18.9 μm for ADM and 134.2 ± 9.8 μm for ACM) (Figure 1G). Additionally, chondrocyte suspensions were quickly absorbed and evenly distributed in both ACM (Figure 1C) and ADM (Figure 1D) scaffolds, which indicated that ACM and ADM had comparable cell affinities. Notably, both ACM and ADM scaffolds maintained their original shape and size after cell seeding. Furthermore, the cell adhesion rate on ACM was lower than that on ADM (88.94 ± 2.17% for ACM and 95.58 ± 2.10% for ADM) (Figure 1F). SEM was used to evaluate ECM production by chondrocytes on ACM and ADM scaffolds during the early stage of *in vitro* culture. The results showed that chondrocytes exhibited a round shape within 24 h, gradually stretched, and eventually secreted ECM to cover the pores in both ACM (Figures 1C1–C3) and ADM (Figures 1D1–D3) scaffolds. Cell viability assays showed that chondrocytes grew well on both ACM and ADM scaffolds with significant proliferation over time and few dead cells were observed at any time point (Figures 2A,B). However, a higher chondrocyte amount and more homogeneous chondrocyte distribution were observed in the ADM group compared with the ACM group, which may have been due to the larger pore size and higher cell adhesion rate. Collectively, these results indicated that both ACM and ADM scaffolds had satisfactory biocompatibility for chondrocytes to attach, proliferate, and produce ECM, whereas the ADM scaffold had advantages, including in a larger pore size and higher cell adhesion rate, compared with ACM scaffolds.

### *In vitro* Engineered Cartilages (ECs)

**FIGURE 3:**
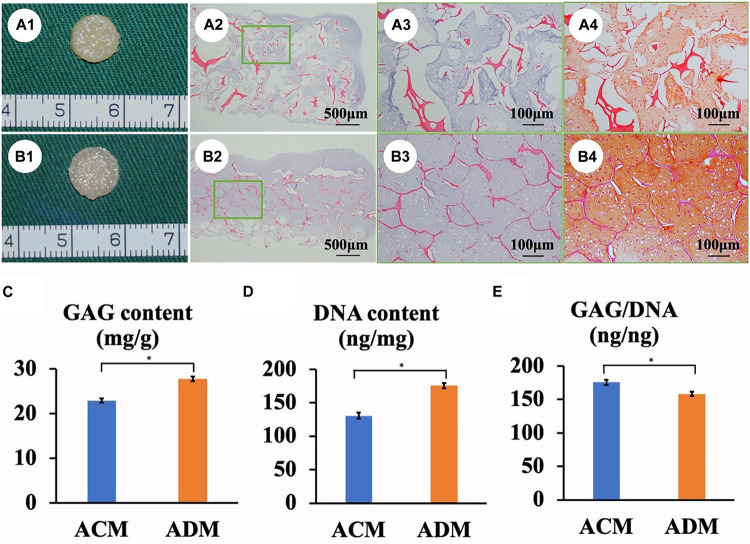
*In vitro* ECs formed by chondrocytes seeded in ACM and ADM scaffolds. Gross view, HE staining, and Safranin-O staining of samples in ACM **(A1–A4)** and ADM **(B1–B4)** groups after 8 weeks of *in vitro* culture. Quantitative analysis of the GAG content **(C)**, DNA content **(D)**, and GAG/DNA ratio **(E)**. *P < 0.05.

**FIGURE 4:**
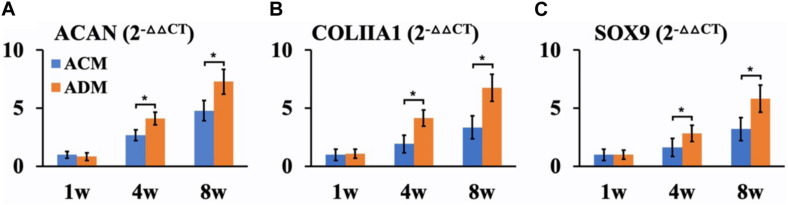
QPCR analysis of *in vitro* ECs in ACM and ADM groups. Expression of ACAN **(A)**, COLIIA1 **(B)**, and SOX9 **(C)** genes in ACM and ADM groups after 1, 4, and 8 weeks of *in vitro* culture. *P < 0.05.

In the current study, *in vitro* ECs maintained their original contour profile during the whole *in vitro* culture course. Although *in vitro* ECs in both ACM and ADM groups exhibited no visible gross differences, histological examinations revealed more ample and homogeneous cartilage-specific ECM in the ADM group (Figures 3B1–B4) compared with the ACM group (Figures 3A1–A4). GAG and DNA contents in the ADM group were significantly higher than those in the ACM group (Figures 3C–E). qPCR revealed that expression of cartilage-related genes, including ACAN, COLIIA1, and SOX9, was significantly higher in the ADM group than in the ACM group at 4 and 8 weeks (Figures 4A–C).

### *In vivo* ECs

**FIGURE 5:**
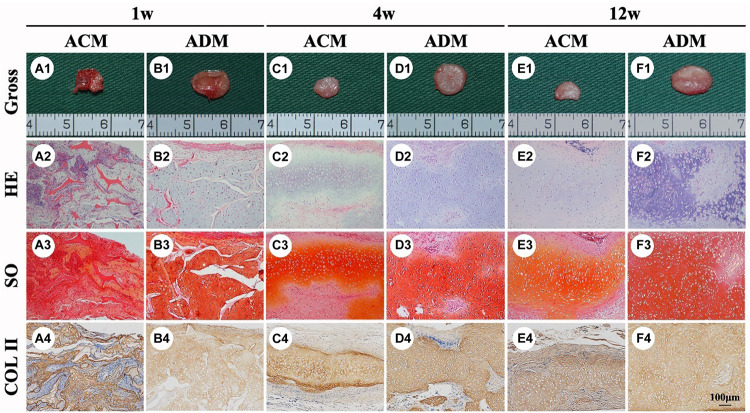
*In vivo* ECs formed by chondrocytes seeded in ACM and ADM scaffolds. Gross view, HE, Safranin-O, and collagen II immunohistochemical staining of ACM **(A1–A4)** and ADM **(B1–B4)** scaffolds after *in vivo* implantation for 1 week. Gross view, HE, Safranin-O, and collagen II immunohistochemical staining of ACM **(C1–C4)** and ADM **(D1–D4)** scaffolds after 4 weeks of *in vivo* implantation. Gross view, HE, Safranin-O, and collagen II immunohistochemical staining of ACM **(E1–E4)** and ADM **(F1–F4)** scaffolds after 12 weeks of *in vivo* implantation.

**FIGURE 6:**
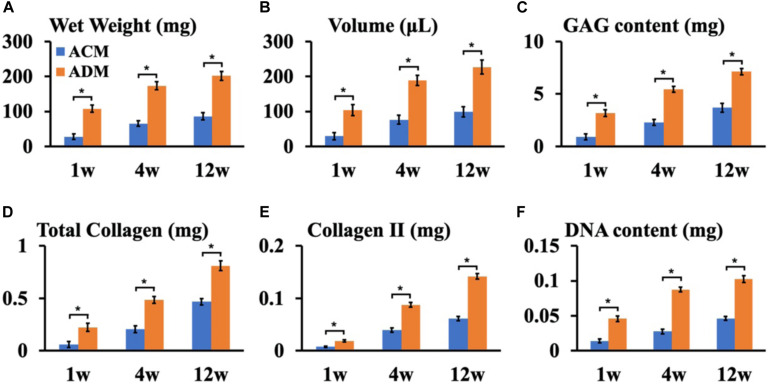
Quantitative analysis of ECs in ACM and ADM groups. Quantitative analysis of the wet weight **(A)**, volume **(B)**, GAG content **(C)**, total collagen **(D)**, collagen II **(E)**, and DNA content **(F)** in ACM and ADM groups after 1, 4, and 12 weeks of *in vivo* implantation. *P < 0.05.

To explore the *in vivo* EC formation abilities of ACM and ADM scaffolds, the above *in vitro* ECs at 8 weeks were subcutaneously implanted into autologous goats. At 1, 4, and 8 weeks after implantation, EC samples from both ACM and ADM groups had gradually matured as evidenced by a reddish appearance at 1 week to an ivory white appearance at 4 and 12 weeks (Figures 5A1–F1). Notably, ACM samples had obviously shrunk during the course of subcutaneous implantation, whereas ADM samples did not shrink or even increased in size. Histology demonstrated that samples in the ACM group had tremendous inflammatory cell infiltration and severe cartilage-specific ECM absorption (Figures 5A2–A4), whereas samples in the ADM group had scarce inflammatory cell infiltration and stable cartilage-specific ECM formation (Figures 5B2–B4). As the implantation time was prolonged to 12 weeks, samples in both ACM and ADM groups exhibited increases in cartilage-specific ECM deposition and typical lacunae structures (Figures 5A2–F2), as observed by positive staining for SO (Figures 5A3–F3) and type II collagen (Figures 5A4–F4). Notably, the histology indicated that scaffolds in both ACM and ADM groups had degraded gradually over the implantation course and had almost completely degraded by 4 weeks after implantation. The quantitative data, including the wet weight, volume, and GAG, total collagen, collagen II, and DNA contents, of both ACM and ADM groups (Figure 6) showed significantly increasing trends with prolongation of the implantation time, which further confirmed that the *in vivo* ECs had matured during the *in vivo* implantation time. Notably, all of these quantitative indexes in the ADM group were significantly higher than those in the ACM group, which indicated that the ADM scaffold had advantages for engineering cartilage compared with the ACM scaffold in terms of subcutaneous implantation.

### Immune Responses

**FIGURE 7:**
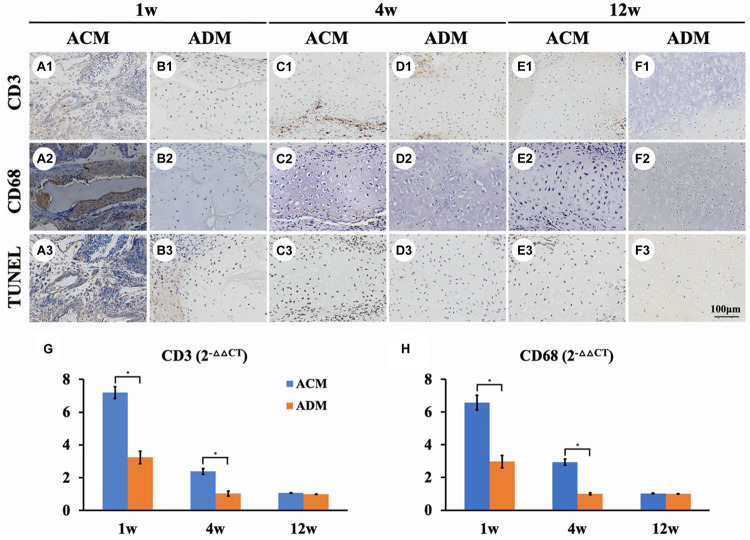
Inflammatory reactions characterized by CD3, CD68, and TUNEL staining. CD3, CD68, and TUNEL staining of *in vivo* ECs in ACM **(A1–A3)** and ADM **(B1–B3)** groups at 1 week after implantation. CD3, CD68, and TUNEL staining of *in vivo* ECs in ACM **(C1–C3)** and ADM **(D1–D3)** groups at 4 weeks after implantation. CD3, CD68, and TUNEL staining of *in vivo* ECs in ACM **(E1–E3)** and ADM **(F1–F3)** groups at 12 weeks after implantation. Expression of CD3 **(G)** and CD68 **(H)** genes in ACM and ADM groups after 1, 4, and 12 weeks of *in vivo* implantation. *P < 0.05.

We further performed immunological and apoptotic examinations to investigate factors that affected *in vivo* cartilage regeneration. After 1 week of subcutaneous implantation, samples in the ACM group showed tremendous inflammatory cell infiltration, especially around the undegradable ACM as indicated by strong positive immunohistochemical staining for CD3 and CD68 as well as TUNEL (Figures 7A1–A3). In stark contrast, scarce inflammatory cell infiltration was observed around the ADM scaffold (Figures 7B1–B3). As the implantation time was prolonged, the inflammatory reaction was significantly reduced in both ACM and ADM groups after 4 weeks of implantation (Figures 7C1–C3,D1–D3), which had almost disappeared as indicated by negative immunohistochemical staining for CD3 and CD68 as well as TUNEL after 12 weeks of implantation (Figures 7E1–E3,F1–F3).

Genes associated with inflammation were analyzed by qPCR to evaluate the intensity of the immune response to xenogeneic materials (Figures 7G,H). Expression levels of inflammation-related genes CD3 and CD68 in ACM samples was much higher than those in ADM samples after 1 week of *in vivo* implantation. The expression levels of CD3 and CD68 in ACM samples at 4 weeks of implantation were still significantly higher than those in ACM samples and almost undetectable after 12 weeks *in vivo*. These results indicated that the inflammatory reaction of ACM was stronger than that of ADM, which might be the most important factor in the absorption of ACM implants.

## Discussion

Increasing attention has been focused on AM materials in tissue engineering because of their intrinsic bioactive components, biomimetic microenvironment, excellent biocompatibility, and suitable biodegradability (Chen et al., 2020b; Xu et al., 2020a). ACM and ADM are the most widely used AMs in engineering cartilage. ACM provides a proper microenvironment for chondrocyte growth and cartilage regeneration in theory because it retains most cartilage-specific structures and functional proteins. ADM is also extensively employed in reconstructing cartilage and has obtained clinical licenses for mature products. In the current study, we confirmed that both ACM and ADM scaffolds generated cartilage in immunocompetent large animals, and ADM had advantages over ACM as a scaffold for cartilage regeneration in terms of a favorable pore structure, homogeneous and stable cartilage regeneration, and low immune response.

Extensively studies have demonstrated that the pore size and three-dimensional structure of scaffolds are crucial factors for cell proliferation, differentiation, and ECM production during cartilage regeneration (Shi et al., 2017; Chen et al., 2020a; Li et al., 2020; Xu et al., 2020b). Although it is generally accepted that ACM is a good scaffold material because of its biomimetic cartilage-specific microenvironment, it is difficult to prepare ACM as a three-dimensional structure with a tunable pore size owing to its poor crosslinking property (Xu et al., 2017; Li et al., 2019). As another AM, ADM has easy accessibility and manufacturing processes, wide resources, and low costs. In this regard, by blending with 30% ADM, we successfully fabricated a porous ACM scaffold using the same protocol that employed a solute, concentration, and freeze drying with pure porous ADM scaffolds. Our study indicated that the pore size of the porous ACM scaffold was significantly smaller than that of its ADM counterpart. The results showed that ADM had advantages over ACM with higher cell adhesion and cell proliferation rates *in vitro*. Additionally, live/dead staining revealed that ACM had a more homogeneous chondrocyte distribution compared with ADM. We speculate that this phenomenon was related the comparatively smaller pore size in the ACM scaffold, which hindered permeability of the high density cell suspension (1 × 10^8^ cells/mL) and the heterogenous pore size in the ACM, scaffold, which may impede chondrocyte attachment, migration, and distribution. The homogeneous chondrocyte distribution of ADM may significantly enhance the quality of regenerated cartilage and give rise to superior neocartilage compared with its ACM counterpart.

We further investigated differences in cartilage regeneration *in vitro* by ACM and ADM scaffolds. As expected, *in vitro* ECs in the ADM group were more homogeneous than those in the ACM group. Additionally, quantitative data, including DNA and GAG contents, confirmed that *in vitro* ECs in the ADM group were superior to those in the ACM group. Furthermore, expression levels of cartilage-specific gene, including SOX9, ACAN, and COL IIA1, in the *in vitro* ECs of the ACM group were lower than those in the *in vitro* ECs of the ADM group at 4 and 8 weeks of culture. Previously studies have indicated that collagen type II triggers a negative feedback loop, which induces the expression of proinflammatory cytokines and matrix metalloproteinases that affect or even destroy production of the ECM (Fichter et al., 2006; Klatt et al., 2009). It is known that the ACM scaffold consists mainly of collagen type II, which might play a feedback inhibitory role in cartilage regeneration.

Achieving stable and homogeneous cartilage regeneration *in vivo*, especially in immunocompetent large animals, is important to determine the prospects of a scaffold material in clinical translation. Our results indicated that abundant inflammatory cells, including lymphocytes and macrophages, had infiltrated around ECs in the ACM group. It is well known that inflammatory cell infiltration can result in chondrocyte apoptosis and even neocartilage absorption (Liu et al., 2016). In stark contrast, samples in the ADM group evoked virtually no inflammatory reactions or apoptosis around ECs, and consequently, gave rise to stable and homogeneous cartilage formation. Notably, scaffolds in the ACM group were completely degraded after 4 weeks of *in vivo* culture accompanied by disappearance of inflammatory reactions and apoptosis, which indicated that the inflammatory reaction was indeed caused by the material. Because the processing of both ACM and ADM scaffolds was identical, the residual cellular content in both the ACM and ADM scaffolds were extremely low, which indicated that the main source of inflammation was not the antigens of xenogeneic tissues, but caused by the two AMs themselves. ADM is derived from dermis and mainly consists of collagen I and III and erect low immune reactions (Liu et al., 1989; Bayrak et al., 2013). In addition, previous study indicated that both the collagen I and III exhibited inhibitive effect in rheumatoid arthritis model (Endler et al., 1978). Our current study also confirmed that the ADM did not induce an inflammatory response or apoptosis in immunocompetent animals except for a moderate inflammatory response and apoptosis at 1 week after implantation, which may have been caused by surgical trauma. It has been proved that collagen II induces proinflammatory cytokines and matrix metalloproteinases *in vivo* (Fichter et al., 2006; Klatt et al., 2009; Shakya and Nandakumar, 2014). Although the ACM scaffold was decellularized to remove antigen-related cellular components, the primitive collagen II was conserved. Our current study confirmed that collagen II-containing ACM caused an immune response and severely impeded cartilage regeneration in immunocompetent large animals. Collectively, our results indicate that ADM is more suitable for cartilage regeneration than ACM and sufficient to generate stable cartilage in immunocompetent large animals.

## Conclusion

We prepared ACM and ADM with three-dimensional porous structures by a freeze-drying method and demonstrated that ADM gives rise to a homogeneous chondrocyte distribution and improves cartilage regeneration compared with its ACM counterpart. Although there are still some mechanisms that need to be explored, the current study indicates that ADM is sufficient to generate stable cartilage in immunocompetent large animals and represents an excellent candidate material for clinical translation of tissue-engineered cartilage.

## Data Availability Statement

The original contributions presented in the study are included in the article/supplementary material, further inquiries can be directed to the corresponding author/s.

## Ethics Statement

The animal study was reviewed and approved by the Weifang Medical University Ethics Committee.

## Author Contributions

YW: scaffolds design, culture cells, and animal operation. YX: data analyses and manuscript revises. GZ: revise. YL: data analyses. YC: funding acquisition and review and editing. All authors contributed to the article and approved the submitted version.

## Conflict of Interest

The authors declare that the research was conducted in the absence of any commercial or financial relationships that could be construed as a potential conflict of interest.
